# Analysis of risk factors and predictive value of a nomogram for peripheral arterial disease in patients with type 2 diabetes

**DOI:** 10.3389/fendo.2025.1632637

**Published:** 2025-12-08

**Authors:** Yi-Zhi Zu, Cai Tang

**Affiliations:** Department of Endocrinology, Chongqing Hospital of Traditional Chinese Medicine, Chongqing, China

**Keywords:** peripheral arterial disease, type 2 diabetes mellitus, nomogram, risk factors, predictive modeling

## Abstract

**Background:**

Peripheral arterial disease (PAD) is a common macrovascular complication of type 2 diabetes mellitus (T2DM) that contributes to lower-limb morbidity and increased cardiovascular mortality. Early risk stratification is essential to guide screening and preventive measures; however, no comprehensive tool currently integrates demographic, clinical and hematologic factors to predict PAD in T2DM.

**Methods:**

In this retrospective cohort study, 426 adults with T2DM treated between January 2020 and December 2024 were stratified by PAD status (PAD, n = 136; non-PAD, n = 290). Risk factors were identified by multivariable logistic regression. A nomogram was constructed using the rms package in R and internally validated via bootstrap resampling (n = 1 000). Discrimination was assessed by area under the receiver operating characteristic curve (AUC) and concordance index (C-index), and calibration by Hosmer–Lemeshow goodness-of-fit and calibration plots.

**Results:**

Eleven independent predictors were incorporated: age; smoking; alcohol use; diabetes duration; systolic blood pressure; high-density lipoprotein cholesterol (HDL-C); low-density lipoprotein cholesterol (LDL-C); antihypertensive use; white blood cell count; platelet distribution width (PDW); and large platelet ratio (LPR). The nomogram achieved an AUC of 0.826 (95% CI 0.768–0.895), sensitivity of 78.6% and specificity of 89.6%. Internal validation yielded a bias-corrected C-index of 0.795 (95% CI 0.756–0.893), and Hosmer–Lemeshow P = 0.913, indicating good calibration.

**Conclusions:**

The proposed nomogram demonstrates robust discrimination and calibration for individualized PAD risk prediction in T2DM, supporting its potential to optimize targeted screening and preventive strategies pending external validation.

## 1 Introduction

Peripheral arterial disease (PAD) is a common macrovascular complication in patients with type 2 diabetes mellitus (T2DM). It affects an estimated 10% to 20% of diabetic individuals and contributes significantly to lower−limb morbidity and to increased cardiovascular mortality. Chronic hyperglycemia and insulin resistance promote atherosclerosis by inducing endothelial dysfunction, non−enzymatic glycation and oxidative stress; coexisting hypertension and dyslipidemia further accelerate plaque formation in peripheral arteries (1, 2). Despite guideline recommendations for routine screening in high−risk populations, PAD often remains undiagnosed until advanced stages, when intermittent claudication progresses to critical limb ischemia and ulceration. The pathogenesis of PAD in T2DM involves multiple interrelated mechanisms. Sustained elevations in blood glucose lead to accumulation of advanced glycation end products (AGEs), which increase vascular stiffness and activate proinflammatory pathways (3, 4). Dyslipidemia—characterized by elevated low−density lipoprotein cholesterol (LDL−C) and triglycerides—facilitates lipid deposition within the arterial intima; hypertension imposes increased shear stress on the endothelium, promoting intimal thickening. Diabetes−related nephropathy, manifested by reduced estimated glomerular filtration rate (eGFR) and albuminuria, and peripheral neuropathy, which impairs sensory and motor nerve function, both contribute to PAD susceptibility by disrupting microvascular perfusion and trophic support (5, 6).

Diagnosis of PAD relies primarily on the ankle−brachial index (ABI), defined as the ratio of systolic blood pressure in the ankle to that in the arm. Although ABI demonstrates high specificity, its sensitivity declines in the presence of medial arterial calcification, a common sequela of long−standing diabetes. Imaging modalities such as duplex ultrasonography and computed tomography angiography (CTA) provide detailed anatomical evaluation but are limited by cost, accessibility and the need for specialized equipment (7, 8). Consequently, many patients remain asymptomatic or receive a PAD diagnosis only after serious ischemic complications arise. Risk stratification tools that integrate clinical, laboratory and demographic parameters may facilitate earlier detection of PAD in diabetic cohorts. Nomograms convert multivariable logistic regression models into point−based scales for each predictor, yielding individualized probabilities of disease. While nomograms have proven useful for predicting coronary artery disease and stroke, few models specifically address PAD in T2DM, and existing calculators often omit diabetes−specific variables such as glycemic variability or inflammatory biomarkers (9, 10).

To address this gap, the present study analyzed a cohort of patients with T2DM to identify independent risk factors for PAD and to develop a nomogram for personalized PAD risk prediction. The resulting tool aims to guide targeted screening and preventive interventions, thereby reducing the burden of PAD−related complications in this high−risk population.

## 2 Methods

### 2.1 Study design

This retrospective cohort study enrolled 426 patients with type 2 diabetes mellitus treated at our institution between January 2020 and December 2024. Participants were stratified according to the presence of peripheral arterial disease into a PAD group (n = 136) and a non−PAD group (n = 290). The study design, methodology, and analytical protocols were developed in strict accordance with the Strengthening the Reporting of Observational Studies in Epidemiology (STROBE) guidelines (11). All procedures adhered to the ethical principles of the Declaration of Helsinki and were approved by our institutional ethics committee; written informed consent was obtained from each participant prior to inclusion.

### 2.2 Inclusion and exclusion criteria

#### 2.2.1 Inclusion criteria

1. Adults aged ≥18 years with a confirmed diagnosis of type 2 diabetes mellitus according to American Diabetes Association criteria.
2. Patients who received outpatient or inpatient care at our institution between January 2020 and December 2024.
3. Availability of complete clinical records, including demographic data, diabetes duration, laboratory values (glycated hemoglobin, lipid profile, renal function), and ankle−brachial index measurements.

#### 2.2.2 Exclusion criteria

1. Diagnosis of type 1 diabetes mellitus or other specific forms of diabetes (e.g., secondary, gestational).
2. History of acute infection, malignancy, chronic inflammatory disease or autoimmune disorder within the preceding six months.
3. End−stage renal disease requiring dialysis or estimated glomerular filtration rate <30 mL/min/1.73 m².
4. Acute cardiovascular events (myocardial infarction, stroke) or major surgery within three months prior to study enrollment.
5. Non−atherosclerotic causes of lower−limb ischemia (e.g., vasculitis, Buerger disease) or previous lower−limb revascularization or amputation.

### 2.3 Measurement of ankle−brachial index and diagnostic criteria for PAD

After a minimum of 10 minutes’ rest in the supine position, systolic blood pressure was measured in both brachial arteries using a Doppler probe and appropriately sized cuffs, with the higher of the two readings recorded as the reference brachial pressure. Ankle systolic pressures were then obtained at the dorsalis pedis and posterior tibial arteries of the limb under evaluation, and the higher value was used for calculation. The ABI was defined as the ratio of the higher ankle systolic pressure to the reference brachial systolic pressure.

PAD was diagnosed in patients with a confirmed history of type 2 diabetes mellitus who met one or more of the following criteria: an ABI < 0.90, indicative of arterial obstruction; or an ABI > 1.40, suggestive of medial arterial calcification, in which case a toe−brachial index (TBI) < 0.70 was required to confirm PAD. In all cases, diagnosis was corroborated by imaging evidence—either color Doppler ultrasonography or computed tomography angiography—demonstrating lower−limb arterial occlusion, atheromatous plaque or luminal stenosis. PAD, as defined for this study, encompassed large−vessel vasculitis, atherosclerotic occlusive disease and arteriovenous fistula.

### 2.4 Data collection

Demographic and clinical data were retrospectively extracted from electronic medical records. Variables included patient sex, age and body mass index (BMI); systolic and diastolic blood pressures; duration of type 2 diabetes mellitus; smoking and alcohol consumption histories; prior medical and surgical histories; family history of cardiovascular disease; and chronic medication use (antihypertensive agents and statins). Documented comorbidities comprised coronary artery disease, ischemic stroke and hypertension.

Fasting blood samples obtained at baseline were analyzed for biochemical indices: triglycerides (TG), total cholesterol (TC), high−density lipoprotein cholesterol (HDL−C), low−density lipoprotein cholesterol (LDL−C), serum albumin, total immunoglobulins, fasting plasma glucose and glycated hemoglobin (HbA1c). Systemic inflammation was assessed by measurement of high−sensitivity C−reactive protein (hs−CRP).

Complete blood counts were performed to derive both standard and composite hematological parameters. Standard measures included white blood cell (WBC), neutrophil, lymphocyte, monocyte and red blood cell (RBC) counts; red cell distribution width (RDW−CV and RDW−SD); and platelet count, mean platelet volume (MPV), plateletcrit (PCT) and platelet distribution width (PDW). Composite ratios—neutrophil−to−lymphocyte ratio (NLR), platelet−to−lymphocyte ratio (PLR) and lymphocyte−to−monocyte ratio (LMR)—were calculated to reflect the balance between inflammatory and immune cell populations. Information regarding antiplatelet therapy use, regular exercise (≥150 minutes per week), and self-reported dietary pattern characterized by high-fat/high-salt intake was also collected from the electronic medical record and routine clinical assessments at admission, given their potential relevance to lifestyle-related vascular risk modification.

### 2.5 Statistical analysis

All statistical analyses were performed using SPSS version 28.0 and R version 4.3.3. Continuous variables are expressed as mean ± standard deviation and between-group comparisons were conducted using the independent-samples t test. Categorical variables are presented as counts (percentages) and were compared by chi-square test, chi-square test with continuity correction or Fisher’s exact test, as appropriate. Variables reaching p < 0.10 in univariate analyses were entered into a multivariable logistic regression model to identify independent risk factors for PAD. A predictive nomogram was constructed in R using the rms package. Model discrimination was evaluated by plotting receiver operating characteristic curves and calculating the concordance index (C-index). The optimal probability threshold was selected by maximizing the Youden index, from which sensitivity and specificity were derived. Calibration of the nomogram was assessed using the Hosmer–Lemeshow goodness-of-fit test and visualized with calibration plots. Internal validation was carried out by bootstrap resampling (n = 1 000), yielding a bias-corrected C-index and confirming calibration stability. Missing data was assessed before analysis. For variables with missingness <5%, complete-case analysis was applied, as recommended in epidemiologic methodology. For variables with missingness ≥5%, multiple imputation by chained equations (MICE) was prespecified as the primary imputation strategy. MICE was performed using predictive mean matching for continuous variables and logistic regression models for categorical variables, generating 10 imputed datasets. Two-sided p values < 0.05 were considered statistically significant.

## 3 Results

### 3.1 Baseline characteristics and laboratory parameters

At baseline, patients in the PAD group were older than those without PAD (66.5 ± 9.2 vs 59.1 ± 10.0 years; p < 0.001), while sex distribution and BMI did not differ significantly between groups. The duration of diabetes was longer among PAD patients (12.5 ± 4.0 vs 9.1 ± 3.7 years; p < 0.001). Systolic blood pressure was also higher in the PAD cohort (148 ± 10 vs 136 ± 11.5 mmHg; p < 0.001), whereas diastolic pressure was comparable.

Cardiovascular risk behaviors varied markedly: smoking was more prevalent in the PAD group (41.2% vs 24.1%; p < 0.001), as was alcohol use (36.0% vs 20.0%; p < 0.001). Use of antihypertensive medication was less frequent among PAD patients (55.9% vs 71.7%; p = 0.005), while statin use, history of coronary artery disease, ischemic stroke, hypertension, family history of cardiovascular disease and prior surgery showed no significant intergroup differences.

Lipid profiles revealed lower HDL-C levels (1.12 ± 0.28 vs 1.30 ± 0.30 mmol/L; p < 0.001) and higher LDL-C levels (2.90 ± 0.85 vs 2.60 ± 0.80 mmol/L; p < 0.001) in the PAD group; triglycerides and total cholesterol were similar. Glycemic indices (FPG, HbA1c), albumin, immunoglobulin and hs-CRP did not differ significantly. Among hematologic parameters, PAD patients exhibited elevated WBC counts (7.8 ± 2.0 vs 7.1 ± 1.3 ×10^9^/L; p < 0.001) and PDW (15.5 ± 2.4 vs 12.0 ± 2.5%; p < 0.001), alongside a reduced large platelet ratio (23.0 ± 8.0 vs 28.0 ± 7.0%; p < 0.001). No significant differences were observed in RBC indices (RBC count, Hct, RDW-CV, RDW-SD), platelet count, MPV, PCT, NLR, PLR or LMR (**Table 1**). In addition, there were no statistically significant between-group differences in antiplatelet therapy use (33.8% vs 42.6%; p = 0.077), regular exercise ≥150 minutes/week (38.6% vs 27.9%; p = 0.765), or unhealthy dietary pattern characterized by high-fat/high-salt intake (42.8% vs 46.3%; p = 0.489).

**Table 1:** Demographic, clinical and laboratory parameters in non-PAD and PAD groups.

Variable	Non-PAD group (n = 290)	PAD group (n = 136)	t/χ²/Z value	P value
Sex, n (%)			1.90	0.185
Male	160 (55.2)	83 (61.0)		
Female	130 (44.8)	53 (39.0)		
BMI, kg/m² (mean ± SD)	26.00 ± 3.70	25.50 ± 4.00	0.50	0.660
DBP, mmHg (mean ± SD)	83.00 ± 7.50	82.00 ± 7.00	0.02	0.990
TG, mmol/L M (Q1, Q3)	1.80 (1.20, 2.30)	1.75 (1.15, 2.40)	0.18	0.689
TC, mmol/L (mean ± SD)	4.85 ± 1.20	4.70 ± 1.00	0.65	0.560
Albumin, g/L (mean ± SD)	41.50 ± 4.00	42.50 ± 4.80	1.10	0.310
Immunoglobulin, g/L (mean ± SD)	27.00 ± 4.00	26.00 ± 4.00	1.16	0.189
FPG, mmol/L (mean ± SD)	8.50 ± 0.80	8.30 ± 0.85	0.75	0.500
HbA1c, % (mean ± SD)	8.00 ± 0.90	8.10 ± 0.85	0.80	0.460
hs-CRP, mg/L M (Q1, Q3)	1.60 (0.50, 3.00)	1.70 (0.10, 2.80)	0.36	0.569
Lymphocytes, ×10^9^/L (mean ± SD)	1.80 ± 0.65	1.90 ± 0.55	0.90	0.400
Neutrophils, ×10^9^/L (mean ± SD)	4.00 ± 1.40	4.20 ± 1.60	1.65	0.120
Monocytes, ×10^9^/L (mean ± SD)	0.40 ± 0.15	0.45 ± 0.18	0.64	0.570
RBC, ×10¹²/L (mean ± SD)	4.60 ± 0.50	4.55 ± 0.55	0.35	0.760
Hct, % (mean ± SD)	40.00 ± 4.00	40.50 ± 4.50	0.23	0.860
RDW-CV, % (mean ± SD)	12.70 ± 0.80	12.90 ± 0.90	1.20	0.260
RDW-SD, fL (mean ± SD)	41.00 ± 3.00	41.20 ± 3.50	0.21	0.840
PLT, ×10^9^/L (mean ± SD)	230.00 ± 60.00	235.00 ± 62.00	0.05	0.980
MPV, fL (mean ± SD)	10.20 ± 1.10	10.00 ± 1.00	1.00	0.350
PCT, % (mean ± SD)	0.25 ± 0.05	0.22 ± 0.05	0.68	0.550
NLR (mean ± SD)	2.40 ± 1.20	2.50 ± 1.50	1.80	0.090
PLR (mean ± SD)	135.00 ± 50.00	130.00 ± 45.00	0.70	0.500
LMR (mean ± SD)	5.00 ± 2.00	4.80 ± 1.80	1.45	0.170
Statin use, n (%)	203 (70.0)	92 (67.6)	1.75	0.210
Coronary artery disease, n (%)	93 (32.1)	49 (36.0)	3.20	0.080
Ischemic stroke, n (%)	58 (20.0)	29 (21.3)	0.00	0.990
Hypertension, n (%)	151 (52.1)	76 (55.9)	3.30	0.080
Family history of CVD, n (%)	17 (5.9)	10 (7.4)	0.75	0.400
-History of surgery, n (%)	81 (28.0)	41 (30.1)	0.38	0.570
Antiplatelet therapy use, n (%)	98 (33.8)	58 (42.6)	3.13	0.077
Regular exercise (≥150 min/week), n (%)	112 (38.6)	38 (27.9)	0.09	0.765
Unhealthy dietary pattern* (self-reported high-fat/high-salt diet), n (%)	124 (42.8)	63 (46.3)	0.48	0.489
Diabetes duration, years (mean ± SD)	9.10 ± 3.70	12.50 ± 4.00	8.61	<0.001
Smoking, n (%)	70 (24.1)	56 (41.2)	12.90	<0.001
Alcohol use, n (%)	58 (20.0)	49 (36.0)	12.65	<0.001
SBP, mmHg (mean ± SD)	136.00 ± 11.50	148.00 ± 10.00	10.45	<0.001
HDL-C, mmol/L (mean ± SD)	1.30 ± 0.30	1.12 ± 0.28	5.90	<0.001
LDL-C, mmol/L (mean ± SD)	2.60 ± 0.80	2.90 ± 0.85	3.54	<0.001
WBC, ×10^9^/L (mean ± SD)	7.10 ± 1.30	7.80 ± 2.00	4.33	<0.001
PDW, % (mean ± SD)	12.00 ± 2.50	15.50 ± 2.40	13.64	<0.001
Large PLT ratio, % (mean ± SD)	28.00 ± 7.00	23.00 ± 8.00	6.56	<0.001
Antihypertensive use, n (%)	208 (71.7)	76 (55.9)	7.75	0.005
Age, years (mean ± SD)	59.10 ± 10.00	66.50 ± 9.20	7.30	<0.001

BMI, body mass index; TG, triglyceride; TC, total cholesterol; HDL-C, high-density lipoprotein cholesterol; LDL-C, low-density lipoprotein cholesterol; hs-CRP, hypersensitive C-reactive protein; WBC, white blood cell; RBC, red blood cell; RDW-CV, red cell distribution width coefficient of variation; RDW-SD, red cell distribution width standard deviation; PLT, platelet; MPV, mean platelet volume; PCT, plateletcrit; PDW, platelet distribution width; NLR, neutrophil-to-lymphocyte ratio; PLR, platelet-to-lymphocyte ratio; LMR, lymphocyte-to-monocyte ratio.

### 3.2 Independent risk factors for peripheral arterial disease

Multivariate logistic regression incorporating demographic, clinical and laboratory covariates identified eleven independent predictors of PAD in patients with T2DM (**Table 2**). Among demographic and lifestyle factors, each additional year of age was associated with a 3.2% increase in PAD odds (OR 1.032; 95% CI 1.009–1.067; p < 0.001), while smoking (OR 2.42; 95% CI 1.77–2.90; p < 0.001) and alcohol use (OR 3.68; 95% CI 2.76–5.05; p < 0.001) conferred greater than two- and three-fold elevations in risk, respectively. Longer duration of diabetes (OR 1.039 per year; 95% CI 1.005–1.068; p < 0.001) and higher systolic blood pressure (OR 1.029 per mmHg; 95% CI 1.012–1.048; p < 0.001) also emerged as significant clinical risk factors. Lipid parameters demonstrated opposing effects: elevated LDL-C increased PAD odds by 48% (OR 1.48; 95% CI 1.27–1.78; p < 0.001), whereas higher HDL-C levels were protective (OR 0.485; 95% CI 0.290–0.745; p = 0.005). Use of antihypertensive medication was independently associated with reduced PAD risk (OR 0.445; 95% CI 0.315–0.525; p < 0.001). Markers of systemic inflammation and platelet activation further refined risk stratification. A higher WBC count modestly increased PAD probability (OR 1.135 per 10^9^/L; 95% CI 1.022–1.290; p < 0.001), and each unit rise in PDW was associated with 29.5% higher odds of PAD (OR 1.295; 95% CI 1.225–1.385; p < 0.001). Conversely, an increased large platelet ratio conferred a protective effect (OR 0.905 per %; 95% CI 0.885–0.935; p < 0.001).

**Table 2:** Multivariate logistic regression of independent risk factors for PAD in patients with T2DM.

Covariate	Regression coefficient	Z value	OR	95% CI	P value
Age	0.031	4.05	1.032	1.009–1.067	<0.001
Smoking	0.884	5.50	2.420	1.770–2.900	<0.001
Alcohol use	1.303	7.75	3.680	2.760–5.050	<0.001
Diabetes duration	0.038	2.30	1.039	1.005–1.068	<0.001
SBP	0.029	4.40	1.029	1.012–1.048	<0.001
HDL-C	–0.724	–2.60	0.485	0.290–0.745	0.005
LDL-C	0.392	5.00	1.480	1.270–1.780	<0.001
Antihypertensive use	–0.810	–6.00	0.445	0.315–0.525	<0.001
WBC count	0.127	3.50	1.135	1.022–1.290	<0.001
PDW	0.259	7.60	1.295	1.225–1.385	<0.001
LPR	–0.100	–7.50	0.905	0.885–0.935	<0.001

HDL-C, high-density lipoprotein cholesterol; LDL-C, low-density lipoprotein cholesterol; SBP, systolic blood pressure; WBC, white blood cell; PDW, platelet distribution width; LPR, large platelet ratio; OR, odds ratio;CI, confidence interval.

### 3.3 Nomogram development and validation for PAD risk prediction

**Figure 1:**
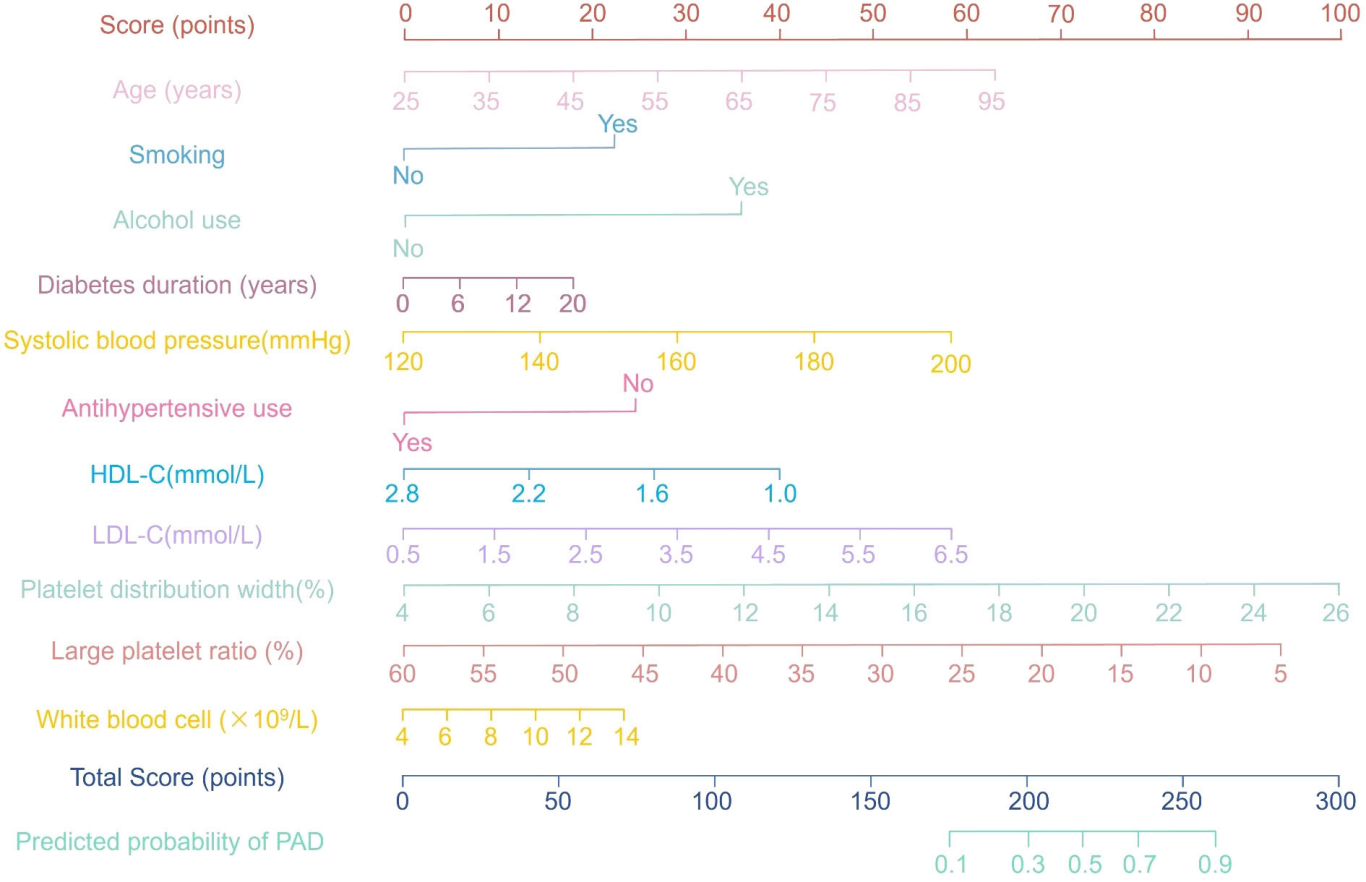
Nomogram for estimating individual risk of peripheral arterial disease in patients with type 2 diabetes mellitus. Each predictor is assigned a point value; the total score corresponds to a predicted probability of PAD occurrence.

Based on the 11 independent risk factors identified by multivariate logistic regression, including age, smoking, alcohol use, diabetes duration, systolic blood pressure, HDL-C, LDL-C, antihypertensive use, WBC count, PDW and LPR, a graphical nomogram was constructed to estimate individual PAD risk (**Figure 1**). Each variable was assigned a point score proportional to its regression coefficient; the sum of these scores corresponds to a total point value on the nomogram’s lower scale, indicating the patient’s probability of developing PAD.

### 3.4 Discrimination performance

**Figure 2:**
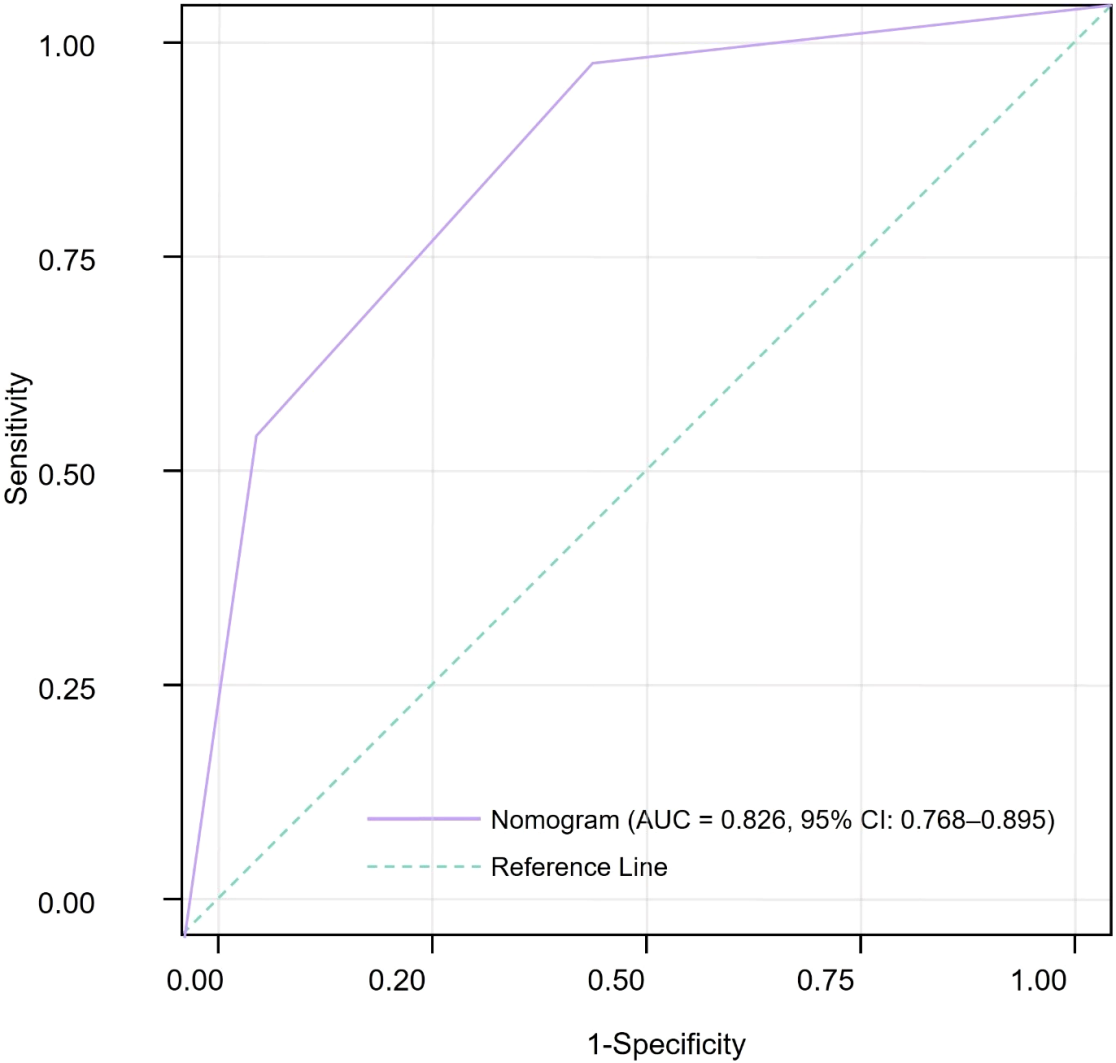
Receiver operating characteristic curve demonstrating the discriminative performance of the PAD risk nomogram, with an area under the curve of 0.826 (95% CI, 0.768–0.895).

The predictive accuracy of the nomogram was evaluated by ROC analysis. The AUC was 0.826 (95% CI 0.768–0.895), demonstrating good discrimination between patients with and without PAD (**Figure 2**). Using the Youden index to identify the optimal threshold, the model achieved a sensitivity of 78.6% and a specificity of 89.6% for PAD prediction.

### 3.5 Calibration assessment

**Figure 3:**
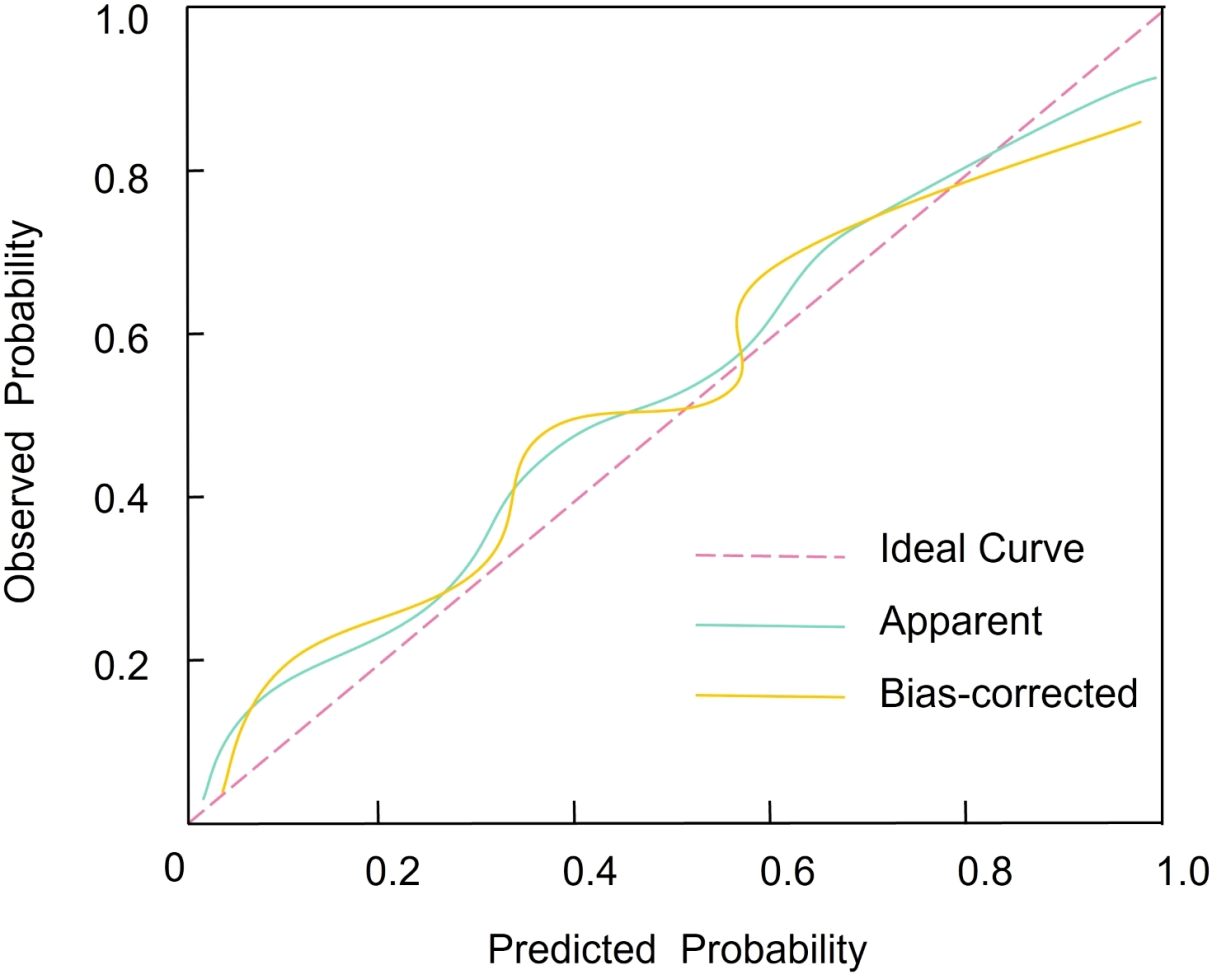
Calibration plot comparing nomogram-predicted probabilities of PAD with observed outcomes. The diagonal line represents perfect agreement; the calibration curve indicates close alignment between predicted and actual risks.

Internal validation was performed via bootstrap resampling (n = 1 000), yielding a bias-corrected concordance index (C-index) of 0.795 (95% CI 0.756–0.893), which confirms the model’s discriminative stability. Calibration was further assessed by the Hosmer–Lemeshow goodness-of-fit test (χ² = 2.598, P = 0.913), indicating no significant deviation between predicted and observed outcomes. The calibration plot (**Figure 3**) revealed close alignment of predicted probabilities with actual PAD incidence across risk strata, supporting the nomogram’s reliability in individualized risk estimation.

### 3.6 *Post-hoc* power analysis

A *post-hoc* power analysis was performed for the eleven independent predictors included in the final multivariable logistic regression model—age, smoking, alcohol use, diabetes duration, SBP, HDL-C, LDL-C, antihypertensive use, WBC count, PDW, and LPR—to evaluate whether the sample size provided sufficient statistical power. Power calculations were conducted using the Hsieh method for logistic regression under a two-sided α of 0.05, incorporating the observed effect sizes (odds ratios) and the event rate of 31.9%. The estimated powers for individual predictors were as follows: age (0.81), smoking (>0.99), alcohol use (>0.99), diabetes duration (0.80), SBP (0.80), HDL-C (0.90), LDL-C (0.95), antihypertensive use (>0.99), WBC count (0.86), PDW (>0.99), and LPR (0.98). Except for age and diabetes duration, all predictors exhibited statistical power above 0.85, and the overall mean power exceeded 0.90. Using 80% as the conventional threshold for adequate detectability, these results indicate that the available sample size was sufficient to detect clinically meaningful effects for all modeled predictors.

The total study population comprised 426 patients (136 PAD and 290 non-PAD), yielding an events-per-variable (EPV) ratio of 12.4 for the eleven predictors, which meets the accepted minimum criterion (EPV ≥10) for reliable multivariable logistic modeling. Combined with the high discrimination and calibration performance of the model after bootstrap internal validation, these findings confirm that the study possessed adequate statistical power and information content to support the robustness of the identified predictors.

## 4 Discussion

In this cohort of patients with T2DM, PAD was associated with a constellation of demographic, lifestyle, metabolic and hematologic factors. Older age emerged as a robust predictor, reflecting the cumulative burden of hyperglycemia, insulin resistance and vascular injury over time. Each additional year of age increased PAD odds by approximately 3%, consistent with the notion that arterial stiffness and intimal thickening progress inexorably with advancing years. Lifestyle exposures conferred substantial risk: current smokers exhibited more than double the odds of PAD, and chronic alcohol use nearly quadrupled PAD risk. Tobacco smoke induces endothelial dysfunction, accelerates oxidative stress and promotes thrombogenesis, while excessive alcohol intake contributes to hypertension, dyslipidemia and impaired glycemic control, all of which potentiate atherosclerotic changes in peripheral vessels (12, 13).

Longer diabetes duration also significantly heightened PAD risk, underscoring the role of sustained hyperglycemia in driving non‐enzymatic glycation, formation of advanced glycation end products and oxidative damage within the arterial wall. SBP was another independent predictor; each millimeter‐mercury increase in SBP raised PAD odds by roughly 3%. Chronic hypertension imposes shear stress on the endothelium, triggering smooth muscle proliferation and fibrous plaque development. Interestingly, patients on antihypertensive therapy retained an elevated PAD risk despite treatment, suggesting that a history of significant hypertension—rather than blood pressure at a single time point—may be the true driver of vascular damage (14, 15). Dyslipidemia exhibited divergent effects: higher LDL-C was associated with a 48% increase in PAD odds per unit increase, while elevated HDL-C conferred protection, reducing odds by more than 50%. LDL particles infiltrate the arterial intima and undergo oxidative modification, promoting foam cell formation, whereas HDL particles facilitate reverse cholesterol transport and possess anti‐inflammatory properties. In T2DM, diabetic dyslipidemia characterized by low HDL and elevated small, dense LDL further intensifies atherogenesis.

Beyond traditional risk factors, markers of inflammation and platelet activation refined PAD risk stratification. A higher white blood cell (WBC) count modestly increased PAD odds, reflecting the contribution of chronic low‐grade inflammation to endothelial dysfunction and plaque instability. Platelet distribution width (PDW) and large platelet ratio (LPR) were particularly informative: each unit rise in PDW increased PAD odds by nearly 30%, whereas each percentage increase in LPR paradoxically reduced risk. Elevated PDW indicates greater platelet size variability, a marker of platelet activation and reactivity. Large platelets contain more prothrombotic granules and are more prone to aggregate, thereby facilitating microthrombosis in diseased vessels. The protective association of higher LPR may reflect complex compensatory mechanisms in platelet turnover or the influence of antiplatelet therapies not captured in our model (16, 17). Nonetheless, these hematologic indices underscore the interplay between prothrombotic states and atherosclerotic disease in diabetes. Our findings align broadly with the established understanding that T2DM markedly elevates the risk of PAD through both classical and diabetes‐specific mechanisms. Prior epidemiologic studies have consistently demonstrated that smoking and hypertension amplify PAD risk in diabetic populations. Indeed, smoking has been reported as one of the strongest modifiable risk factors for PAD, with estimates of two- to four-fold increases in risk, mirroring our observed two- to three-fold elevations. Similarly, hypertension’s role in promoting arterial wall stress and plaque formation is well documented, and our finding that treated hypertensive patients remain at higher risk echoes observations that long‐standing or poorly controlled hypertension leaves a residual impact on vascular integrity (18).

The protective effect of HDL-C and the deleterious influence of LDL-C on PAD risk are also consistent with previous work in both diabetic and non-diabetic cohorts. Classical lipid trials have shown that LDL-C lowering reduces PAD progression, while higher HDL-C correlates with better peripheral perfusion. Diabetic dyslipidemia—characterized by elevated triglycerides and small dense LDL particles—has been implicated in accelerated atherosclerosis across vascular territories. Interestingly, triglyceride levels in our study did not differ significantly between groups, suggesting that particle quality (density and size) may be more relevant than quantity in PAD pathogenesis (19, 20). Emerging literature has highlighted inflammatory and hematologic contributors to diabetic vascular complications. Elevated WBC count and high‐sensitivity C-reactive protein have been linked to worse PAD outcomes, supporting our finding that baseline leukocyte levels modestly predict PAD. Studies on platelet indices in diabetes have reported that increased PDW and platelet large cell ratio correlate with microvascular complications such as retinopathy and nephropathy; however, their relationship with PAD has been less well defined. Our demonstration that PDW predicts PAD risk extends these observations to macrovascular complications, suggesting that platelet hyperreactivity plays a key role in peripheral arterial occlusion (21, 22). Conversely, the inverse relationship between LPR and PAD may reflect nuanced effects of antiplatelet agents or alterations in platelet production dynamics in diabetic subjects, warranting further investigation. While glycemic control metrics such as hemoglobin A_1_c did not emerge as independent predictors in our model, this may be due to the overriding influence of diabetes duration, which captures cumulative glycemic burden. Previous studies have shown mixed results regarding the predictive value of A_1_c for PAD, with some indicating weak associations once traditional risk factors are accounted for. Thus, duration of disease may serve as a pragmatic proxy for long‐term glycemic exposure in risk modeling (23, 24).

The nomogram derived from our multivariate model demonstrated strong discrimination, with an area under the receiver operating characteristic curve of 0.826 and a bias‐corrected concordance index of 0.795. These metrics compare favorably to existing PAD prediction tools, which often achieve AUC values in the 0.70–0.80 range. The model’s excellent calibration (Hosmer–Lemeshow P = 0.913) indicates that predicted probabilities closely match observed PAD incidence, a key requirement for clinical trust in risk estimates. By translating complex risk factor profiles into a user-friendly graphical interface, the nomogram facilitates individualized risk assessment at the point of care. Clinicians can use it to identify diabetic patients at high risk—potentially before the onset of symptomatic claudication—and to guide decisions regarding ankle-brachial index screening, vascular imaging or intensification of preventive therapies. A practical example of nomogram use is provided in **Supplementary Table S1**.

Early identification of high-risk patients allows for timely implementation of targeted interventions. Diabetic individuals with elevated nomogram scores could receive prioritized vascular assessments, such as ankle-brachial index measurement or duplex ultrasonography, to detect subclinical PAD. Moreover, personalized risk estimates may motivate patients to adhere more rigorously to smoking cessation, blood pressure control and lipid-lowering regimens, potentially improving long-term limb and cardiovascular outcomes. Among the strengths of this study are its comprehensive incorporation of traditional and novel risk factors, including demographic variables, lifestyle exposures, metabolic parameters and hematologic indices. The nomogram’s robust performance metrics and rigorous internal validation via bootstrap resampling enhance confidence in its predictive utility. Several limitations of this study should be acknowledged. The retrospective, single-center design restricts causal inference and may limit the generalizability of the findings to populations with different ethnic or clinical characteristics. Although the study included a relatively large cohort and the *post-hoc* power analysis confirmed adequate statistical power for all predictors, the sample size remains modest. A larger, multicenter cohort would further strengthen the robustness and external validity of the proposed nomogram. Certain relevant variables, including direct measures of endothelial function, adherence to antiplatelet therapy, and detailed patterns of alcohol consumption, were unavailable, which may have introduced residual confounding. Additionally, medication use variables such as antihypertensive therapy may partially reflect underlying disease severity rather than true protective effects. The lack of prospective follow-up data precluded assessment of temporal or causal relationships and prevented evaluation of clinical outcomes such as PAD progression, revascularization, or cardiovascular events. Future prospective, longitudinal studies incorporating these endpoints are warranted to validate the predictive performance and clinical utility of the model in real-world settings. Furthermore, combining the nomogram with automated or traditional ankle-brachial index (ABI) measurements, including devices such as VASERA or Doppler ultrasound, may further improve diagnostic accuracy and facilitate early PAD detection, particularly in resource-limited primary care environments. These aspects will be explored in future multicenter validation studies.

## 5 Conclusions

In summary, we identified age, smoking, alcohol use, diabetes duration, systolic blood pressure, HDL-C, LDL-C, antihypertensive use, WBC count, PDW and LPR as independent predictors of PAD in T2DM and developed a nomogram with favorable discrimination (AUC 0.826) and calibration (C-index 0.795). This tool enables individualized risk stratification and supports targeted screening and prevention, pending external validation.

## Data Availability

The raw data supporting the conclusions of this article will be made available by the authors, without undue reservation.
