# Supplementary material for: Analysis of risk factors and predictive value of a nomogram for peripheral arterial disease in patients with type 2 diabetes
Source: Front Endocrinol (Lausanne). 2025 Dec 8;16:1632637. doi: 10.3389/fendo.2025.1632637 (PMC12719262; doi:10.3389/fendo.2025.1632637)
Supplement: Supplementary file 1 [file Table1.docx]

**Supplementary Table S1. Example of nomogram use**

**Example patient**

Male with the following profile: age 50 y; smoking Yes; alcohol use Yes; diabetes duration 15 y; SBP 160 mmHg; antihypertensive use Yes; HDL-C 1.6 mmol/L; LDL-C 1.5 mmol/L; PDW 14 %; LPR 40 %; WBC 8 ×10⁹/L.

| **Predictor** | **Patient value** | **Points (approx.)** |
| --- | --- | --- |
| Age | 50 y | 23 |
| Smoking | Yes | 22 |
| Alcohol use | Yes | 36 |
| Diabetes duration | 15 y | 17 |
| SBP | 160 mmHg | 28 |
| Antihypertensive use | Yes | 0 |
| HDL-C | 1.6 mmol/L | 25 |
| LDL-C | 1.5 mmol/L | 8 |
| PDW | 14% | 43 |
| LPR | 40% | 33 |
| WBC | 8 ×10⁹/L | 8 |
| **Total** | — | **243** |

**Risk readout.** A **Total Score of 243** corresponds, on the nomogram’s bottom scale, to a **predicted probability of PAD ≈ 0.80** (≈80%).

**Interpretation.** This patient’s estimated PAD risk is high by the nomogram. Clinical use should consider this probability together with the optimal probability threshold derived in the main analysis (Youden index; see Section 3.4), as well as patient preferences and guideline-directed care.

**Note.** Points above are approximate visual readings from the printed nomogram. Exact probabilities can be reproduced from the underlying logistic model coefficients; however, the nomogram is intended for routine bedside estimation.
